# E-Cigarette Nicotine Delivery Among Young Adults by Nicotine Form, Concentration, and Flavor: A Crossover Randomized Clinical Trial

**DOI:** 10.1001/jamanetworkopen.2024.26702

**Published:** 2024-08-09

**Authors:** Yoo Jin Cho, Toral Mehta, Alice Hinton, Ruth Sloan, Jean Nshimiyimana, Alayna P. Tackett, Megan E. Roberts, Marielle C. Brinkman, Theodore L. Wagener

**Affiliations:** 1 Center for Tobacco Research, The Ohio State University Comprehensive Cancer Center, Columbus; 2 Division of Health Behavior & Health Promotion, College of Public Health, The Ohio State University, Columbus; 3 Division of Epidemiology, College of Public Health, The Ohio State University, Columbus; 4 Division of Medical Oncology, College of Medicine, The Ohio State University, Columbus

## Abstract

**Importance:**

Concerns have been raised about the abuse liability of modern e-cigarettes that use acidic additives to form nicotine salts, making the inhalation of nicotine smoother than freebase nicotine.

**Objective:**

To examine the effects of nicotine form and concentration and e-liquid flavor on subjective effects ratings, vaping behavior, and nicotine uptake among young adults who use e-cigarettes.

**Design, Setting, and Participants:**

In this single-blind, within-participant, crossover randomized clinical trial, a convenience sample of young adults aged 21 to 25 years who currently used e-cigarettes was recruited from December 2021 to August 2023, for in-person research laboratory visits in Columbus, Ohio.

**Interventions:**

Participants completed up to 9 vaping sessions, starting with their usual e-cigarette brand in the first session followed by 1 of 8 laboratory-prepared e-liquids in a randomly assigned order in each subsequent session. Prepared e-liquids varied by nicotine form (salt-based vs freebase), nicotine concentration (5% vs 1% weight per weight), and flavor (menthol vs tobacco). Each session included a 5-minute, 10-puff standardized vaping period followed by 30 minutes of ad libitum vaping.

**Main Outcomes and Measures:**

At 4 time points (0, 5, 10, and 35 minutes) during each vaping session, plasma samples were collected for assessing nicotine uptake, and self-reports of urges, craving, and withdrawal were collected via questionnaires. Positive subjective effects were self-reported after 35 minutes of vaping using a visual analog scale; urges and cravings were reported using the Questionnaire of Smoking Urges (QSU). Puff topography data were collected throughout each vaping session.

**Results:**

Seventy-two participants (mean [SD] age, 22.4 [1.4] years; 42 [58.3%] female) who sampled at least 1 laboratory-prepared e-liquid composed the analytic sample. Salt-based (vs freebase) nicotine e-liquids increased nicotine intake, with 5% salt-based e-liquids delivering the highest mean plasma levels of nicotine (11.2 ng/mL [95% CI, 9.3-13.2 ng/mL] at 5 minutes; 17.2 ng/mL [95% CI, 14.3-20.1 ng/mL] at 35 minutes) irrespective of flavors. Higher positive subjective effect ratings (eg, for liking) were received by salt-based (42.8; 95% CI, 39.4-46.1) vs freebase (32.0; 95% CI, 28.6-35.3) nicotine, 1% (43.4; 95% CI, 40.2-46.6) vs 5% (31.2; 95% CI, 27.7-34.6) nicotine, and menthol-flavored (43.2; 95% CI, 39.7-46.7) vs tobacco-flavored (31.5; 95% CI, 28.4-34.7) e-liquids. Salt-based and 1% but not menthol-flavored nicotine elicited more intense puffing (eg, 25% [95% CI, 12%-40%] more total puffs for nicotine salts vs freebase). All study e-liquids reduced urges and cravings, with 5% vs 1% nicotine being more effective (mean [SE] QSU-Desire score at 35 minutes, 15.4 [0.5] vs 16.7 [0.5]).

**Conclusions and Relevance:**

In this crossover randomized clinical trial among young adult e-cigarette users, salt-based (vs freebase) nicotine e-liquids increased nicotine intake and yielded more positive subjective effects ratings and intense puffing behaviors, suggesting higher abuse potential. Restricting the level of acidic additives and menthol flavoring may reduce the addictiveness of e-cigarettes.

**Trial Registration:**

ClinicalTrials.gov Identifier: NCT05458895

## Key Points

**Question:**

Do salt-based nicotine and menthol flavoring additives in e-cigarettes increase abuse potential among young adults?

**Findings:**

In this crossover randomized clinical trial of 72 participants aged 21 to 25 years who used e-cigarettes, salt-based (vs freebase) nicotine resulted in higher nicotine intake after both 5-minute standardized and 30-minute ad libitum vaping, particularly at 5% (vs 1%) nicotine. Compared with freebase nicotine, nicotine salts yielded more positive subjective effects ratings and intense puffing behaviors, and menthol (vs tobacco) flavor yielded more positive subjective effects ratings.

**Meaning:**

The findings imply that salt-based nicotine formulations prevalent in the market may increase nicotine dependence among young adults already using e-cigarettes and warrant regulation.

## Teaser

This crossover randomized clinical trial examines effects of nicotine form, concentration, and e-liquid flavor on nicotine uptake and self-reported craving and subjective experiences among young adults who use e-cigarettes.

## Introduction

E-cigarettes have disproportionately attracted youths and are the most commonly used tobacco product among young people in the US.^1,2,3,4,5,6,7^ According to the most recent available data, 18% of young adults in 2021^8^ and 10% of middle and high school students in 2023^7^ reported current e-cigarette use. Young people are not merely experimenting with e-cigarettes but are increasingly becoming established users, with nearly half (46%) of young adults and a quarter (25%) of adolescents who use e-cigarettes reporting daily use.^7,8^ It has been hypothesized that nicotine dependence in young people has increased because of the market transitioning from harsher freebase nicotine to more palatable nicotine salts.^9,10,11^ While e-cigarettes may serve as a safer alternative to regular cigarettes for adults who smoke^12,13,14^ and may have reduced smoking among youths,^15^ studies on the effect of e-cigarette characteristics, including nicotine form, on abuse liability are urgently needed to address the high rates of e-cigarette use among young people.

Compared with freebase nicotine, nicotine salts are easier to inhale and increase the addiction potential of e-cigarettes.^11^ While first-generation e-liquids contained freebase nicotine and were perceived as harsh, an e-cigarette manufacturer reduced the level of freebase nicotine by 90%, mirroring the tobacco industry tactic of manipulating the pH of cigarette smoke to enhance nicotine delivery and palatability.^16,17,18^ This innovation enabled the marketing of e-cigarettes with a nicotine concentration approximately 3 times higher than that of traditional freebase nicotine e-cigarettes, maximizing nicotine delivery.^19^ Accordingly, in a market dominated by these high-nicotine products, use of e-cigarettes among adolescents increased rapidly in 2018^20^ and peaked at 28% in 2019.^6^

Emerging evidence indicates that nicotine salt–based e-cigarettes are more addictive than those with freebase nicotine.^9,10,11,21^ Observational data show increased self-reported nicotine dependence coinciding with the market domination of salt-based nicotine e-cigarettes.^9,10^ In randomized clinical trials, nicotine salts were associated with increased palatability,^11^ puff duration, and mouth-level nicotine exposure during standardized puffing sessions.^21^ However, the evidence during ad libitum puffing sessions is mixed,^21,22,23^ with no difference observed in product liking or nicotine pharmacokinetics across varying levels of salt-based nicotine.^22,23^

Previous research has largely focused on the significant association of various e-liquid flavors and e-cigarette marketing with youth vaping,^24,25^ leading to restrictions on sales and marketing of flavored pod-style e-cigarettes.^26^ Salt-based nicotine and menthol flavor additives have remained in pod and other types of e-cigarette products, although no menthol-flavored e-cigarettes have been authorized to be legally marketed. It is well established that menthol in cigarettes masks the harshness of nicotine and is associated with increased nicotine dependence,^27,28^ but menthol in e-cigarettes warrants further examination to understand its abuse liability.^29,30,31,32^

Using a single-blind, randomized, within-participant, crossover design, this experimental study examined the effects of nicotine salts (vs freebase nicotine) on the abuse liability of e-cigarettes in young adults who exclusively use e-cigarettes. Participants engaged in both standardized and ad libitum puffing, and e-liquids were varied in nicotine concentration (5% vs 1% weight per weight) and e-liquid flavor (menthol vs tobacco). Given previous research suggesting that adults who have never smoked perceive nicotine salt as smoother than those who have,^11^ it was hypothesized that nicotine salts (vs freebase nicotine), high (vs low) nicotine concentration, and menthol (vs tobacco) flavor would result in higher nicotine uptake, more positive subjective effect ratings, and more intense vaping behavior.

## Methods

### Participants

In this crossover randomized clinical trial (NCT05458895), young adults aged 21 to 25 years who currently used e-cigarettes were recruited to complete in-person research laboratory visits in Columbus, Ohio. Eligible participants were those who (1) had used e-cigarettes daily for the past 3 months, (2) were willing to abstain from nicotine products for at least 12 hours, and (3) were willing to complete the study protocol in either 5 or 9 laboratory visits, depending on participant choice. Exclusion criteria were (1) self-reported diagnosis of lung disease, cardiac event, or cardiac distress within the past 3 months; (2) current or planned pregnancy or breastfeeding; (3) using other tobacco products or cannabis on 10 days or more in the past month; or (4) attempting to quit vaping. Participants were enrolled from December 2021 to August 2023. Race and ethnicity, ascertained by self-report, were collected for descriptive purposes and were not included in the statistical analysis; categories were Hispanic, non-Hispanic Black (hereafter, *Black*), non-Hispanic White (hereafter, *White*), and other (included non-Hispanic American Indian, non-Hispanic Asian, non-Hispanic Pacific Islander, and multiracial). The study was approved by The Ohio State University cancer institutional review board and followed the Consolidated Standards of Reporting Trials (CONSORT) reporting guideline.^33^ Each participant provided written informed consent during the first laboratory visit. The trial protocol is available in Supplement 1.

### Design and Materials

A single-blind, randomized, within-participant, crossover design was used, in which each participant completed up to 9 vaping sessions. In the first session, each participant vaped their own e-cigarette brand, and in each of the subsequent 8 sessions, they vaped 1 of 8 prefilled study e-liquids in random order using the same study e-cigarette device (Evolv Reflex [Evolv LLC]) (eFigure 1 in Supplement 2) held at a constant wattage (8 W). e-Liquids were prepared to differ only by nicotine concentration (1% vs 5% weight per weight), nicotine form (freebase vs salt-based nicotine), and flavor (menthol vs tobacco). The ingredients and analysis of the e-liquids are provided in eTables 1 and 2, respectively, in Supplement 2.

### Procedure

Eligible individuals were identified via an online screener survey linked to social media advertisements. Eligibility was verified via telephone, the participants’ baseline visit was scheduled, and they were asked to abstain from nicotine for at least 12 hours before their visit. All visits involved the collection of exhaled carbon monoxide, blood samples, and for individuals who were biologically female, a urine sample for a pregnancy test. Participants were informed that their 12-hour nicotine abstinence would be confirmed during the visits through blood plasma nicotine analysis, which was a bogus pipeline.^34^ Participants who reported a minimum of 12-hour abstinence from nicotine, had exhaled carbon monoxide levels of 10 ppm or lower, and were not pregnant proceeded with the vaping sessions.

Participants completed vaping sessions in a ventilated smoking room. During the baseline visit, lasting up to 3 hours, each participant first used their own e-cigarette device and e-liquid for a single vaping session. After the baseline visit, participants received a study e-cigarette device prefilled with a randomly assigned study e-liquid for practice at home until their second visit. Laboratory visits were each separated by at least 48 hours and preceded by 12-hour nicotine abstinence. Given the significant time commitment required for study completion, each participant chose to complete the remaining 8 vaping sessions in either 4 or 8 laboratory visits (eFigure 2 in Supplement 2) per protocol amendment in August 2022. Subsequent visits lasted up to 6 hours for the 4-visit option, with a 3-hour washout period between vaping sessions to allow nicotine levels to return to baseline, and up to 2 hours for the 8-visit option (eTable 3 in Supplement 2). The study e-liquids were administered in a random sequence generated by a statistician (A.H.) and remained blinded to participants. Each vaping session consisted of a directed 5-minute, 10-puff vaping period (1 puff every 30 seconds) immediately followed by 30 minutes of ad libitum vaping.

### Outcome Measures

Plasma nicotine level (ng/mL) was assessed by collecting 3-mL venous blood samples at 4 time points (0, 5, 10, and 35 minutes) for each vaping session. Plasma was separated using a centrifuge and stored at −80 °C prior to analysis.

Subjective effects of study products were self-reported after each vaping session using a visual analog scale (range, 0-100, with higher ratings indicating more positive subjective effects) adapted from a drug effects and liking questionnaire.^35^ Questions included 5 items assessing the positive subjective effects of wanting, liking, enjoyment, pleasure, and satisfaction (eg, “How much do you like the study product?”).

Puffing topography data were collected in real time using the SPA-D Smoking Puff Analyzer (Sodim) during the ad libitum vaping sessions. The data comprised total puff count, puff duration (seconds), interpuff interval (seconds), volume per puff (mL), total puff volume (mL), and mean puff velocity (mL/s).^36^

Urges and cravings for vaping were self-reported at 4 time points (0, 5, 10, and 35 minutes) for each vaping session using the Questionnaire of Smoking Urges (QSU-Brief)^37^ with a 7-point scale (higher score indicates greater craving) and the 8-item Minnesota Nicotine Withdrawal Scale (MNWS; score range, 0-4; higher score indicates greater withdrawal symptoms).^38^ Two subscales of the QSU-Brief, each consisting of 5 items on desire (QSU-Desire) and 11 items on relief (QSU-Relief), were used for the analysis (score range, 1-35; higher score indicates greater desire or lower relief). For the MNWS, a total score (MNWS-Total; range, 0-32; higher score indicates greater overall withdrawal severity) and a score for an item on craving (MNWS-Craving; range, 0-4; higher score indicates greater craving) were used.

### Statistical Analysis

The primary analysis used generalized linear mixed models (GLMMs) to examine the associations of nicotine form (nicotine salt vs freebase nicotine), nicotine concentration (1% vs 5%), and flavor (menthol vs tobacco) with outcome measures of plasma nicotine level, subjective effects, puffing topography, and nicotine craving. Each GLMM included random participant effects and fixed effects for nicotine form, concentration, and flavor while controlling for the minimum time abstinent since the last e-liquid was sampled (3 hours or 12 hours). Additional analyses included an interaction between nicotine form and concentration, and Tukey adjustment for multiple comparisons was used to compare conditions. Plasma nicotine level, topography measures, and craving measures were log transformed for analysis. Effect estimates with corresponding 95% CIs are reported; for all log-transformed outcomes, effect estimates are represented as percentage change for interpretability.

Potential carryover and period effects were explored by estimating the interactions of the given e-liquid with the preceding e-liquid and the given e-liquid with the treatment order. Analyses were conducted using SAS, version 9.4 (SAS Institute Inc). All statistical tests were 2-sided, and *P* <  .05 was considered statistically significant.

## Results

### Participant Characteristics

A total of 87 participants were included in the study (51 [58.6%] female, 36 [41.4%] male; mean [SD] age, 22.4 [1.4] years). Of these, 4 (4.6%) were Black; 6 (6.9%), Hispanic; 68 (78.2%), White; and 9 (10.3%), other race and ethnicity or multiracial. Seventy-two participants (82.8%) who attended their second laboratory visit and used at least 1 study e-liquid composed the analytic sample. Their mean (SD) age was 22.4 (1.4) years; 3 (4.2%) were Black; 6 (8.3%), Hispanic; 55 (76.4%), White; and 8 (11.1%), other race and ethnicity or multiracial. A total of 42 (58.3%) were female, and 30 (41.7%) were male. These participants reported a mean (SD) of 27 (6.2) days of e-cigarette use in the past 30 days, with minimal use of other tobacco products (ie, fewer than 10 days in the past month), and did not significantly differ in sociodemographics or tobacco use history with the 15 participants (17.2%) who attended only the first visit and did not use study e-liquids (eTable 4 in Supplement 2). The CONSORT diagram of participant flow through the trial is provided as eFigure 5 in Supplement 2.

### Plasma Nicotine Level

**Figure 1.**
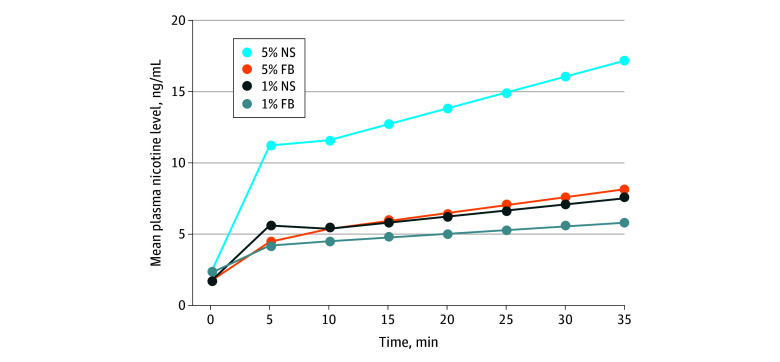
Plasma Nicotine Levels by Nicotine Form and Concentration The 35-minute vaping session comprised 5 minutes of standardized puffing followed by 30 minutes of ad libitum puffing. FB indicates freebase nicotine; NS, nicotine salts.

Significant main effects on nicotine delivery were observed for nicotine form, concentration, and flavor. Nicotine salt (vs freebase nicotine) resulted in 94% (95% CI, 74%-115%) higher plasma nicotine levels after 5 minutes of standardized vaping and 63% (95% CI, 48%-80%) higher levels after an additional 30 minutes of ad libitum vaping. Higher nicotine concentration (5% vs 1%) yielded 49% (95% CI, 34%-65%) higher plasma nicotine levels at 5 minutes and 65% (95% CI, 49%-81%) higher levels at 35 minutes. Participants using an e-liquid with menthol flavor (vs tobacco) showed no significant difference (4%; 95% CI, −6% to 15%) in plasma nicotine levels at 5 minutes but had 18% (95% CI, 7%-30%) higher levels at 35 minutes (Table 1). Additional analysis revealed a significant interaction between nicotine form and concentration, with 5% salt-based nicotine delivering the highest mean levels of nicotine (11.2 ng/mL [95% CI, 9.3-13.2 ng/mL] at 5 minutes [*P* < .001]; 17.2 ng/mL [95% CI, 14.3-20.1 ng/mL] at 35 minutes [*P* = .002]) (Figure 1).

**Table 1.** Unadjusted Mean Levels of Plasma Nicotine by Nicotine Form, Concentration, and Flavor at 4 Time Points

Variable	Plasma nicotine level, mean (95% CI), ng/mL			
	0 min	5 min	10 min	35 min
Nicotine form				
Salt	2.0 (1.6-2.4)	8.4 (7.3-9.5)^a^	8.5 (7.3-9.6)^a^	12.3 (10.7-14)^a^
Freebase	2.0 (1.4-2.5)	4.3 (3.7-4.9)^a^	4.9 (4.3-5.5)^a^	7.0 (6.1-7.9)^a^
Nicotine concentration, %				
1	2.0 (1.4-2.5)	4.9 (4.3-5.5)^a^	4.9 (4.4-5.5)^a^	6.7 (5.9-7.4)^a^
5	2.0 (1.5-2.4)	7.8 (6.7-9.0)^a^	8.5 (7.3-9.6)^a^	12.6 (10.9-14.3)^a^
Flavor				
Menthol	1.9 (1.3-2.4)	6.6 (5.5-7.7)	7.1 (6.0-8.1)	10.2 (8.7-11.6)^a^
Tobacco	2.1 (1.7-2.5)	6.1 (5.4-6.9)	6.3 (5.5-7.1)	9.1 (7.8-10.4)^a^

^a^ Significant difference between the 2 conditions of nicotine form, concentration, and flavor in generalized linear mixed models.

### Subjective Effects

Nicotine salt (vs freebase nicotine), 1% nicotine concentration (vs 5%), and menthol (vs tobacco) flavor all resulted in higher ratings for wanting, liking, enjoyment, pleasure, and satisfaction (Table 2). For example, mean ratings for liking were 42.8 (95% CI, 39.4-46.1) for salt-based vs 32.0 (95% CI, 28.6-35.3) for freebase nicotine, 43.4 (95% CI, 40.2-46.6) for 1% vs 31.2 (95% CI, 27.7-34.6) for 5% nicotine, and 43.2 (95% CI, 39.7-46.7) for menthol-flavored vs 31.5 (95% CI, 28.4-34.7) for tobacco-flavored e-liquids.

**Table 2.** Unadjusted Means of Subjective Effect Ratings by Nicotine Form, Concentration, and Flavor at 35 Minutes

Variable	Rating, mean (95% CI)^a^				
	Wanting	Liking	Enjoyment	Pleasure	Satisfaction
Nicotine form					
Salt	38.8 (35.6-41.9)	42.8 (39.4-46.1)	43.3 (40.0-46.6)	45.2 (41.9-48.4)	45.6 (42.4-48.8)
Freebase	29.5 (26.5-32.5)	32.0 (28.6-35.3)	32.3 (29.0-35.7)	32.8 (29.5-36.0)	35.4 (32.3-38.5)
Nicotine concentration, %					
1	38.8 (35.8-41.8)	43.4 (40.2-46.6)	43.9 (40.7-47.1)	44.3 (41.2-47.4)	43.2 (40.1-46.3)
5	29.3 (26.1-32.5)	31.2 (27.7-34.6)	31.5 (28.2-34.9)	33.4 (30.0-36.8)	37.6 (34.3-41.0)
Flavor					
Menthol	38.9 (35.7-42.1)	43.2 (39.7-46.7)	43.2 (39.8-46.7)	44.3 (40.9-47.7)	44.9 (41.6-48.1)
Tobacco	29.4 (26.4-32.3)	31.5 (28.4-34.7)	32.4 (29.2-35.6)	33.6 (30.5-36.7)	36.1 (33.0-39.2)

^a^ Ratings were on a scale of 0 to 100, with higher ratings indicating more positive subjective effects. All estimates showed a significant difference between the 2 conditions of nicotine form, concentration, and flavor in generalized linear mixed models.

**Figure 2.**
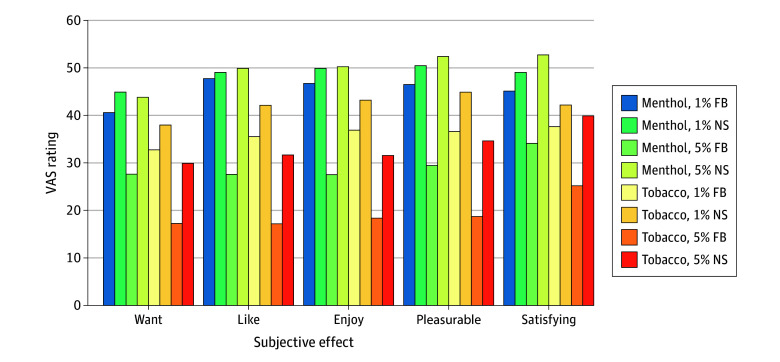
Mean Ratings of Subjective Effects FB indicates freebase nicotine; NS, nicotine salts; VAS, visual analog scale (range, 0-100, with higher ratings indicating more positive subjective effects).

There was a significant interaction between nicotine form and concentration (*P* < .001) for all 5 measures. Nicotine salt (vs freebase nicotine) was more positively rated for all 5 measures for 5% nicotine but not for 1% nicotine. As shown in Figure 2, tobacco-flavored 5% freebase nicotine was rated lowest for all 5 measures, and menthol-flavored 5% nicotine salts were rated highest for 4 of the 5 measures (like, enjoy, pleasurable, and satisfying).

### Puffing Topography

Nicotine salt (vs freebase nicotine) resulted in more intense puffing, yielding 25% (95% CI, 12%-40%) more total puffs, 5% (95% CI, 2%-8%) longer mean puff duration, 11% (95% CI, 6%-16%) larger mean volume per puff, and 9% (95% CI, 0%-20%) larger total puff volume. Nicotine form did not significantly effect mean interpuff interval or puff velocity (Table 3).

**Table 3.** Unadjusted Means of Puffing Topography Measures by Nicotine Form, Concentration, and Flavor

Variable	Measure, mean (95% CI)					
	Total puff count, No.	Puff duration, s	Interpuff interval, s	Volume per puff, mL	Total puff volume, mL	Velocity, mL/S
Nicotine form						
Salt	16.0 (14.4-17.6)^a^	3.0 (2.8-3.1)^a^	117.7 (106.3-129.1)	46.4 (43.5-49.3)^a^	785.2 (702.4-868.1)^a^	16.8 (16.0-17.7)
Freebase	14.2 (12.6-15.8)^a^	2.9 (2.8-3.0)^a^	123.9 (109.8-138.0)	42.2 (40.1-44.4)^a^	742.3 (664.2-820.4)^a^	17.7 (16.6-18.8)
Nicotine concentration, %						
1	17.1 (15.5-18.7)^a^	3.1 (3.0-3.3)^a^	115.1 (102.6-127.5)^a^	49.4 (46.6-52.2)^a^	900.6 (813.5-987.6)^a^	17.3 (16.5-18.2)
5	13.1 (11.6-14.6)^a^	2.7 (2.6-2.8)^a^	126.9 (114.0-139.8)^a^	38.7 (36.8-40.7)^a^	608.8 (543.8-673.8)^a^	17.2 (16.1-18.3)
Flavor						
Menthol	15.2 (13.7-16.8)	3.0 (2.8-3.1)^a^	114.8 (103.7-125.8)	44.8 (42.5-47.1)	757.3 (684.9-829.7)	16.9 (16.1-17.8)
Tobacco	15.0 (13.3-16.6)	2.9 (2.8-3.1)^a^	126.6 (112.4-140.7)	43.9 (41.1-46.6)	773.1 (683.8-862.3)	17.5 (16.5-18.6)

^a^ Significant difference between the 2 conditions of nicotine form, concentration, and flavor in generalized linear mixed models.

Lower nicotine concentration (1% vs 5%) led to more intense puffing, resulting in a 47% (95% CI, 31%-65%) increase in total puff count, 20% (95% CI, 17%-24%) longer mean puff duration, 15% (95% CI, 7%-22%) lower mean interpuff interval, 25% (95% CI, 20%-31%) larger mean volume per puff, and 54% (95% CI, 41%-68%) larger total puff volume, with no effect on mean puff velocity. Menthol (vs tobacco) flavor resulted in 4% (95% CI, 1%-7%) longer mean puff duration, but flavors did not have a significant effect on any of the other topography measures.

There was a significant interaction between nicotine form and concentration for total puff count (*P* = .002), mean volume per puff (*P* = .008), and mean puff duration (*P* = .001). Nicotine salt (vs freebase nicotine) yielded higher puff count, volume per puff, and duration for 5% nicotine but not 1% nicotine (all *P* < .001). No interaction effect was found for the remaining 3 topography measures. eFigure 3 in Supplement 2 shows each puffing topography measure by nicotine form and concentration.

### Urges and Cravings

All study e-liquids effectively reduced cravings, with significantly lower ratings for QSU-Desire, QSU-Relief, MNWS-Total, and MNWS-Craving at 5, 10, and 35 minutes compared with baseline (eFigure 4 in Supplement 2). Neither nicotine form nor flavor impacted urges and cravings after 35 minutes of vaping. Lower nicotine concentration (1% vs 5%) was less effective in reducing cravings at 35 minutes, resulting in 8% (95% CI, 3%-14%) higher scores for QSU-Desire (mean [SE], 16.7 [0.5] vs 15.4 [0.5]) and 4% (95% CI, 1%-8%) higher scores for QSU-Relief (mean [SE], 9.5 [0.3] vs 8.9 [0.3]); nicotine concentration was not found to significantly effect either MNWS-Total or MNWS-Craving scores.

There was a significant interaction between nicotine form and concentration for QSU-Desire (*P* = .01), such that lower nicotine concentration (1% vs 5%) reduced desire less effectively in nicotine salt but not in freebase nicotine formulations. However, no significant interaction was present for QSU-Relief, MNWS-Total, or MNWS-Craving (eFigure 4 in Supplement 2).

## Discussion

The findings of this crossover randomized clinical trial suggest that the shift from harsher freebase nicotine e-liquids to more palatable nicotine salts in e-cigarettes increased factors associated with nicotine dependence among young adult users. The study provides data showing that nicotine salts are more addictive than freebase nicotine, demonstrating consistent results across various abuse liability measures: nicotine uptake in both standardized and ad libitum vaping sessions (Table 1), subjective effects (Table 2), and puffing topography (Table 3). The results extend prior findings that nicotine salts made e-cigarettes more palatable and increased puff duration and mouth-level nicotine exposure.^11,21^

In this study, nicotine salts paired with a 5% nicotine concentration—as is common in modern e-cigarettes in the US—exhibited a particularly high abuse liability, achieving the highest plasma nicotine levels irrespective of flavors (Figure 1). These levels met or exceeded the nicotine delivery of a combustible cigarette (approximately 15 ng/mL in 10-12 puffs^39^), averaging 11.2 ng/mL after 5 minutes of standardized vaping and 17.2 ng/mL after 30 minutes of ad libitum vaping. Compared with 5% freebase nicotine, 5% nicotine salts were rated more positively across all 5 subjective effect items (Figure 2), suggesting that nicotine salts offset the harsh physiological effect of high nicotine concentrations and may have enabled the tobacco industry to increase nicotine concentrations without compromising product appeal.^18^

While nicotine salts are associated with an increased risk of dependence on e-cigarettes among youths,^11^ their improved nicotine delivery and palatability may have enhanced the effectiveness of e-cigarettes in helping people who smoke cigarettes to quit. This notion is supported by continually mounting evidence from both observational studies^13,14^ and randomized clinical trials.^12^ As such, future studies should identify a minimum threshold for the fraction of freebase nicotine in e-cigarettes, which could minimize their appeal for young nonsmokers while maintaining them as a safer alternative for smokers.^40^

The findings demonstrate that menthol is another factor that likely increases e-cigarette abuse liability, consistent with previous studies indicating that menthol improves the sensory experience of vaping.^29,30,31,32^ For example, menthol-flavored 5% nicotine salts were rated more positively than tobacco-flavored 5% nicotine salts across all 5 subjective effects (Figure 2). While menthol did not result in higher nicotine uptake following 5 minutes of standardized vaping, a difference was observed following an additional 30 minutes of ad libitum vaping. Taken together, the present findings suggest that restricting the level of nicotine salts^11,41^ and menthol as a characterizing flavor in e-liquids could reduce the appeal and abuse liability of e-cigarettes for young adults.

Many countries have proposed or imposed restrictions on nicotine concentration in e-cigarettes to 2%.^42,43,44,45^ Yet, focusing solely on nicotine concentrations is a limited approach. In this study, lower nicotine concentrations were rated more positively for subjective effects (Table 2) and were associated with compensatory puffing (Table 3), indicating a larger overall exposure to e-cigarette aerosol.^39,46,47^ Furthermore, using e-cigarettes with low nicotine concentrations may lead users to use devices with higher power to increase nicotine delivery, potentially exposing them to higher levels of toxicants.^48^ Hence, while lower nicotine concentrations reduced nicotine delivery in the current study’s laboratory setting, regulations should consider other e-cigarette characteristics, such as device power and puffing topography.

### Strengths and Limitations

One strength of this study is that the e-liquids differed only by the parameters of interest (ie, nicotine form, concentration, and flavor), and thus, differences can be directly associated with these manipulations. The study also used a more comprehensive approach than prior studies in which participants were exposed to either only a single puff^11^ or an online ad libitum puffing session^22^ by incorporating both standard and ad lib puffing sessions.

Despite these strengths, the study has limitations. First, the findings may not generalize to people who smoke, given that nicotine salts and freebase nicotine showed no difference in nicotine pharmacokinetics, subjective effects, and puffing topography among people who smoke.^22,23^ The participants also may not represent all e-cigarette users, including those who frequently use other nicotine products or cannabis. Second, the study was not double-blinded, which may have potentially introduced minor bias by the staff. Third, the e-cigarette device used in this study may not be reflective of what young adults use in naturalistic settings.

## Conclusions

In this crossover randomized clinical trial, salt-based (vs freebase) nicotine e-liquids increased nicotine intake and yielded more positive subjective effects ratings and intense puffing behaviors. Also, menthol (vs tobacco) flavor yielded more positive subjective effects ratings. The findings suggest that nicotine salts play a significant role in increasing nicotine dependence among young people who vape, exacerbated by the market entry of e-cigarettes with higher nicotine concentrations and the availability of menthol-flavored e-liquids that make the vaping experience positive. Developing e-cigarette product standards that restrict the levels of nicotine salts and menthol additives in e-liquids may reduce the abuse liability of e-cigarettes and potentially the development of nicotine dependence among young people.

## Notes

Supplement 1.
Trial Protocol

Supplement 2. **eTable 1.** Ingredients of the Investigational Tobacco Products **eTable 2.** Theoretical and Measured Ingredients in the Investigational Tobacco Products **eTable 3.** Participant Characteristics and Tobacco Use History by Preferred Number of Visits **eTable 4.** Participant Characteristics and Tobacco Use History **eFigure 1.** Study Device **eFigure 2.** Study Sequence **eFigure 3.** Topography During the 30-Minute Ad Libitum Vaping Session by Nicotine Concentration and Form **eFigure 4.** Urges and Cravings for Vaping **eFigure 5.** CONSORT Diagram
eReference

Supplement 3.
Data Sharing Statement

## References

[1] Cornelius ME, Loretan CG, Jamal A, . Tobacco product use among adults—United States, 2021. MMWR Morb Mortal Wkly Rep. 2023;72(18):475-483. doi:10.15585/mmwr.mm7218a1 37141154 PMC10168602

[2] Cornelius ME, Loretan CG, Wang TW, Jamal A, Homa DM. Tobacco product use among adults—United States, 2020. MMWR Morb Mortal Wkly Rep. 2022;71(11):397-405. doi:10.15585/mmwr.mm7111a1 35298455 PMC8942309

[3] Cornelius ME, Wang TW, Jamal A, Loretan CG, Neff LJ. Tobacco product use among adults—United States, 2019. MMWR Morb Mortal Wkly Rep. 2020;69(46):1736-1742. doi:10.15585/mmwr.mm6946a4 33211681 PMC7676638

[4] Creamer MR, Wang TW, Babb S, . Tobacco product use and cessation indicators among adults—United States, 2018. MMWR Morb Mortal Wkly Rep. 2019;68(45):1013-1019. doi:10.15585/mmwr.mm6845a2 31725711 PMC6855510

[5] Wang TW, Asman K, Gentzke AS, . Tobacco product use among adults—United States, 2017. MMWR Morb Mortal Wkly Rep. 2018;67(44):1225-1232. doi:10.15585/mmwr.mm6744a2 30408019 PMC6223953

[6] Wang TW, Gentzke AS, Creamer MR, . Tobacco product use and associated factors among middle and high school students—United States, 2019. MMWR Surveill Summ. 2019;68(12):1-22. doi:10.15585/mmwr.ss6812a1 31805035 PMC6903396

[7] Birdsey J, Cornelius M, Jamal A, . Tobacco product use among US middle and high school students—National Youth Tobacco Survey, 2023. MMWR Morb Mortal Wkly Rep. 2023;72(44):1173-1182. doi:10.15585/mmwr.mm7244a1 37917558 PMC10629751

[8] Erhabor J, Boakye E, Obisesan O, . E-cigarette use among US adults in the 2021 Behavioral Risk Factor Surveillance System Survey. JAMA Netw Open. 2023;6(11):e2340859. doi:10.1001/jamanetworkopen.2023.40859 37921768 PMC10625038

[9] Hammond D, Reid JL. Trends in vaping and nicotine product use among youth in Canada, England and the USA between 2017 and 2022: evidence to inform policy. Tob Control. Published online November 8, 2023. doi:10.1136/tc-2023-058241 37940402 PMC11610498

[10] Glantz S, Jeffers A, Winickoff JP. Nicotine addiction and intensity of e-cigarette use by adolescents in the US, 2014 to 2021. JAMA Netw Open. 2022;5(11):e2240671. doi:10.1001/jamanetworkopen.2022.40671 36342713 PMC9641541

[11] Leventhal AM, Madden DR, Peraza N, . Effect of exposure to e-cigarettes with salt vs free-base nicotine on the appeal and sensory experience of vaping: a randomized clinical trial. JAMA Netw Open. 2021;4(1):e2032757. doi:10.1001/jamanetworkopen.2020.32757 33433597 PMC7804919

[12] Hartmann-Boyce J, Lindson N, Butler AR, . Electronic cigarettes for smoking cessation. Cochrane Database Syst Rev. 2022;11(11):CD010216. 36384212 10.1002/14651858.CD010216.pub7 PMC9668543

[13] Kasza KA, Tang Z, Seo YS, . Divergence in cigarette discontinuation rates by use of Electronic Nicotine Delivery Systems (ENDS): longitudinal findings from the United States PATH Study waves 1-6. Nicotine Tob Res. Published online April 3, 2024. doi:10.1093/ntr/ntae027 38566367 PMC11750739

[14] Kasza KA, Edwards KC, Kimmel HL, . Association of e-cigarette use with discontinuation of cigarette smoking among adult smokers who were initially never planning to quit. JAMA Netw Open. 2021;4(12):e2140880. doi:10.1001/jamanetworkopen.2021.40880 34962556 PMC8715340

[15] Levy DT, Warner KE, Cummings KM, . Examining the relationship of vaping to smoking initiation among US youth and young adults: a reality check. Tob Control. 2019;28(6):629-635. doi:10.1136/tobaccocontrol-2018-054446 30459182 PMC6860409

[16] Stevenson T, Proctor RN. The secret and soul of Marlboro: Phillip Morris and the origins, spread, and denial of nicotine freebasing. Am J Public Health. 2008;98(7):1184-1194. doi:10.2105/AJPH.2007.121657 18511721 PMC2424107

[17] Keithly L, Ferris Wayne G, Cullen DM, Connolly GN. Industry research on the use and effects of levulinic acid: a case study in cigarette additives. Nicotine Tob Res. 2005;7(5):761-771. doi:10.1080/14622200500259820 16191747

[18] Duell AK, Pankow JF, Peyton DH. Nicotine in tobacco product aerosols: “It’s déjà vu all over again.” Tob Control. 2020;29(6):656-662. 31848312 10.1136/tobaccocontrol-2019-055275 PMC7591799

[19] Prochaska JJ, Vogel EA, Benowitz N. Nicotine delivery and cigarette equivalents from vaping a JUULpod. Tob Control. 2022;31(e1):e88-e93. doi:10.1136/tobaccocontrol-2020-056367 33762429 PMC8460696

[20] Gentzke AS, Creamer M, Cullen KA, . Vital signs: tobacco product use among middle and high school students—United States, 2011-2018. MMWR Morb Mortal Wkly Rep. 2019;68(6):157-164. doi:10.15585/mmwr.mm6806e1 30763302 PMC6375658

[21] Talih S, Hanna E, Salman R, . Influence of nicotine form and nicotine flux on puffing behavior and mouth-level exposure to nicotine from electronic nicotine delivery systems. Drug Alcohol Depend. 2024;254:111052. doi:10.1016/j.drugalcdep.2023.111052 38103538 PMC10872307

[22] Pauwels CGGM, Visser WF, Pennings JLA, . Sensory appeal and puffing intensity of e-cigarette use: influence of nicotine salts versus free-base nicotine in e-liquids. Drug Alcohol Depend. 2023;248:109914. doi:10.1016/j.drugalcdep.2023.109914 37245418

[23] Frosina J, McEwan M, Ebajemito J, . Assessing the impact of protonating acid combinations in e-cigarette liquids: a randomised, crossover study on nicotine pharmacokinetics. Sci Rep. 2023;13(1):10563. doi:10.1038/s41598-023-37539-6 37386281 PMC10310785

[24] Notley C, Gentry S, Cox S, . Youth use of e-liquid flavours—a systematic review exploring patterns of use of e-liquid flavours and associations with continued vaping, tobacco smoking uptake or cessation. Addiction. 2022;117(5):1258-1272. doi:10.1111/add.15723 34784651 PMC9299186

[25] Wang Y, Duan Z, Weaver SR, . Association of e-cigarette advertising, parental influence, and peer influence with US adolescent e-cigarette use. JAMA Netw Open. 2022;5(9):e2233938. doi:10.1001/jamanetworkopen.2022.33938 36173633 PMC9523494

[26] FDA finalizes enforcement policy on unauthorized flavored cartridge-based e-cigarettes that appeal to children, including fruit and mint. News release. Food and Drug Administration; January 2, 2020. Accessed March 5, 2024. https://www.fda.gov/news-events/press-announcements/fda-finalizes-enforcement-policy-unauthorized-flavored-cartridge-based-e-cigarettes-appeal-children

[27] Lee YO, Glantz SA. Menthol: putting the pieces together. Tob Control. 2011;20(Suppl_2)(suppl 2):ii1-ii7. doi:10.1136/tc.2011.043604 21504926 PMC3085012

[28] Yerger VB. Menthol’s potential effects on nicotine dependence: a tobacco industry perspective. *Tob Control*. 2011;20(Suppl 2):ii29-ii36. 10.1136/tc.2010.041970 PMC3088468 21504929

[29] Tackett AP, Han DH, Peraza N, . Effects of “ice” flavoured e-cigarettes with synthetic cooling agent WS-23 or menthol on user-reported appeal and sensory attributes. Tob Control. Published online November 8, 2023. doi:10.1136/tc-2023-058125 37940405 PMC11076411

[30] Rosbrook K, Green BG. Sensory effects of menthol and nicotine in an e-cigarette. Nicotine Tob Res. 2016;18(7):1588-1595. doi:10.1093/ntr/ntw019 26783293 PMC4902888

[31] DeVito EE, Jensen KP, O’Malley SS, . Modulation of “protective” nicotine perception and use profile by flavorants: preliminary findings in e-cigarettes. Nicotine Tob Res. 2020;22(5):771-781. doi:10.1093/ntr/ntz057 30995302 PMC7368338

[32] Leventhal A, Cho J, Barrington-Trimis J, Pang R, Schiff S, Kirkpatrick M. Sensory attributes of e-cigarette flavours and nicotine as mediators of interproduct differences in appeal among young adults. Tob Control. 2020;29(6):679-686. doi:10.1136/tobaccocontrol-2019-055172 31852818 PMC7473634

[33] Dwan K, Li T, Altman DG, Elbourne D. CONSORT 2010 statement: extension to randomised crossover trials. BMJ. 2019;366:l4378. doi:10.1136/bmj.l4378 31366597 PMC6667942

[34] Roese NJ. Twenty years of bogus pipeline research: a critical review and meta-analysis. Psychol Bull. 1993;114(2):363-375. doi:10.1037/0033-2909.114.2.363

[35] Zacny JP, Conley K, Marks S. Comparing the subjective, psychomotor and physiological effects of intravenous nalbuphine and morphine in healthy volunteers. J Pharmacol Exp Ther. 1997;280(3):1159-1169. 9067299

[36] Lee EM, Malson JL, Waters AJ, Moolchan ET, Pickworth WB. Smoking topography: reliability and validity in dependent smokers. Nicotine Tob Res. 2003;5(5):673-679. doi:10.1080/1462220031000158645 14577984

[37] Cox LS, Tiffany ST, Christen AG. Evaluation of the Brief Questionnaire of Smoking Urges (QSU-Brief) in laboratory and clinical settings. Nicotine Tob Res. 2001;3(1):7-16. doi:10.1080/14622200020032051 11260806

[38] Hughes JR, Hatsukami D. Signs and symptoms of tobacco withdrawal. Arch Gen Psychiatry. 1986;43(3):289-294. doi:10.1001/archpsyc.1986.01800030107013 3954551

[39] Wagener TL, Floyd EL, Stepanov I, . Have combustible cigarettes met their match? the nicotine delivery profiles and harmful constituent exposures of second-generation and third-generation electronic cigarette users. Tob Control. 2017;26(e1):e23-e28. doi:10.1136/tobaccocontrol-2016-053041 27729564 PMC5574194

[40] Cho YJ, Brinkman MC, Hinton A, . The sweet spot study—developing e-liquid product standards for nicotine form and concentration to improve public health: protocol for a randomized, double-blinded, crossover study. PLoS One. 2023;18(9):e0291522. doi:10.1371/journal.pone.0291522 37699050 PMC10497122

[41] Han DH, Wong M, Peraza N, Dose–response effects of two nicotine salt formulations on electronic cigarette appeal and sensory attributes. *Tob Control*. Published online January 2, 2023. 10.1136/tc-2022-057553 PMC10314953 36593119

[42] Health Canada. Vaping products—new limits on nicotine concentration and consultation on flavour restrictions. June 2021. https://www.canada.ca/en/health-canada/news/2021/06/backgrounder-vaping-products--new-limits-on-nicotine-concentration-and-consultation-on-flavour-restrictions.html

[43] European Commission. Electronic cigarettes. 2014. https://health.ec.europa.eu/tobacco/product-regulation/electronic-cigarettes_en

[44] The National Archives of the UK Government. The Tobacco and Related Products Regulations 2016. UK Statutory Instruments 2016, No 507, part 6, regulation 36. Accessed March 5, 2024. https://www.legislation.gov.uk/uksi/2016/507/regulation/36/made

[45] Aaron DG, Wallace CR, Sinha MS. Including e-cigarettes in the FDA rule limiting nicotine. JAMA. 2023;330(12):1129-1130. doi:10.1001/jama.2023.14254 37639253

[46] Leavens ELS, Wagener TL. E-cigarettes and FDA nicotine cap. JAMA. 2024;331(4):358-359. doi:10.1001/jama.2023.24521 38261053 PMC11201304

[47] Hoetger C, Bono RS, White AM, Barnes AJ, Cobb CO. The interaction of nicotine concentration and device power on Electronic Nicotine Delivery System (ENDS) abuse liability among exclusive ENDS users and dual users of ENDS and combustible cigarettes. Exp Clin Psychopharmacol. 2022;30(6):973-982. doi:10.1037/pha0000523 34647773 PMC9284402

[48] Talih S, Salman R, El-Hage R, Might limiting liquid nicotine concentration result in more toxic electronic cigarette aerosols? *Tob Control*. 2021;30(3):348-350. 10.1136/tobaccocontrol-2019-055523 PMC9281877 32522818

