# Supplementary material for: E-Cigarette Nicotine Delivery Among Young Adults by Nicotine Form, Concentration, and Flavor: A Crossover Randomized Clinical Trial
Source: JAMA Netw Open. 2024 Aug 9;7(8):e2426702. doi: 10.1001/jamanetworkopen.2024.26702 (PMC11316233; doi:10.1001/jamanetworkopen.2024.26702)
Supplement: Supplement 1. — Trial Protocol [file jamanetwopen-e2426702-s001.pdf]

STUDY PROTOCOL

ADMINISTRATIVE INFORMATION

The study, entitled "Evaluating E-Cigarette Nicotine Form, Concentration, and Flavors Among Youth," is registered in ClinicalTrials.gov with the Identifier NCT05458895. It was funded by the American Heart Association (YVNR35490079). The principal investigators are Theodore L. Wagener, PhD, the Director of the Center for Tobacco Research and co-leader of the Cancer Control Program at Ohio State University (OSU)'s Comprehensive Cancer Center, as well as a Professor in the Department of Internal Medicine, and Marielle C. Brinkman, Research Professor in OSU's College of Public Health.

INTRODUCTION

A. Background

The Tobacco Control Act grants the US FDA the authority to set "product standards"—including those "respecting the construction, components, ingredients, additives, constituents and properties" of tobacco products, if it can establish that such standard would be "appropriate for the protection of the public health."[1] One possible standard that has the potential to reduce e-cigarette appeal and continued use, particularly among youth and non-tobacco users, is a restriction on the allowable proportion of nicotine in the protonated form (i.e., nicotine salts) in e-liquid. To provide regulators and public health officials with the evidence needed for the development of an effective e-liquid product standard, this study will conduct an experimental study examining how the manipulation of nicotine form (nicotine salt (NSB) vs. free-base (FB)) influences vaping behavior, abuse liability, toxicant exposure and heart and lung health. We will also examine if flavors may weaken the benefit of a nicotine form product standard. Our team has conducted five studies examining NSB e-cig devices,[2-4] including human laboratory studies.[5,6] Results of these studies demonstrated that NSB e-cigs can deliver cigarette-like levels of nicotine,[5,6] have the potential for significant levels of addiction,[2-4] and that lower e-liquid pH was associated with reduced perceived harshness of the aerosol ($p<.05$), increased nicotine uptake ($p<.05$), and increased puffing flow rates and average puff volumes ($ps<.05$).

B. Objectives

Utilizing two human laboratory studies, this study will examine the influence of nicotine form and concentration, and e-liquid flavor on youth vaping behavior, nicotine uptake, abuse liability, toxicant exposure, and acute cardiovascular and pulmonary effects. H1a: Nicotine salt (vs. free-base) and menthol flavored e-liquids (vs. tobacco flavored) will result in puffs of longer duration and higher flow rate, and a greater total inhaled aerosol volume, and H1b: demonstrate greater nicotine uptake, abuse liability, and adverse cardiovascular and pulmonary effects. H1c: High concentration, free-base nicotine (vs. salt nicotine) will result in puffs of shorter duration, lower flow rate, less volume, and lower abuse liability.

C. Trial design

Utilizing a within-subjects, factorial design, 60 e-cig users (aged 21-25 years) will complete vaping sessions which will include a standardized, 5-minute, 10-puff vaping bout (30 seconds between each puff) followed by 30 minutes of ad libitum vaping. This study will include Option A consisting of 9 lab visits each approximately 2 hours long, except visit 1, which will last up to 3 hours OR Option B consisting of 5 lab visits each containing 2 vaping sessions separated by a

3-hour washout period and lasting approximately 6 hours (Figure 1). Figure 2 illustrates the study e-cig and e-liquid variations. Figure 3 depicts the sequence of each vaping session.

Figure 1. Study Sequence: Options A and B

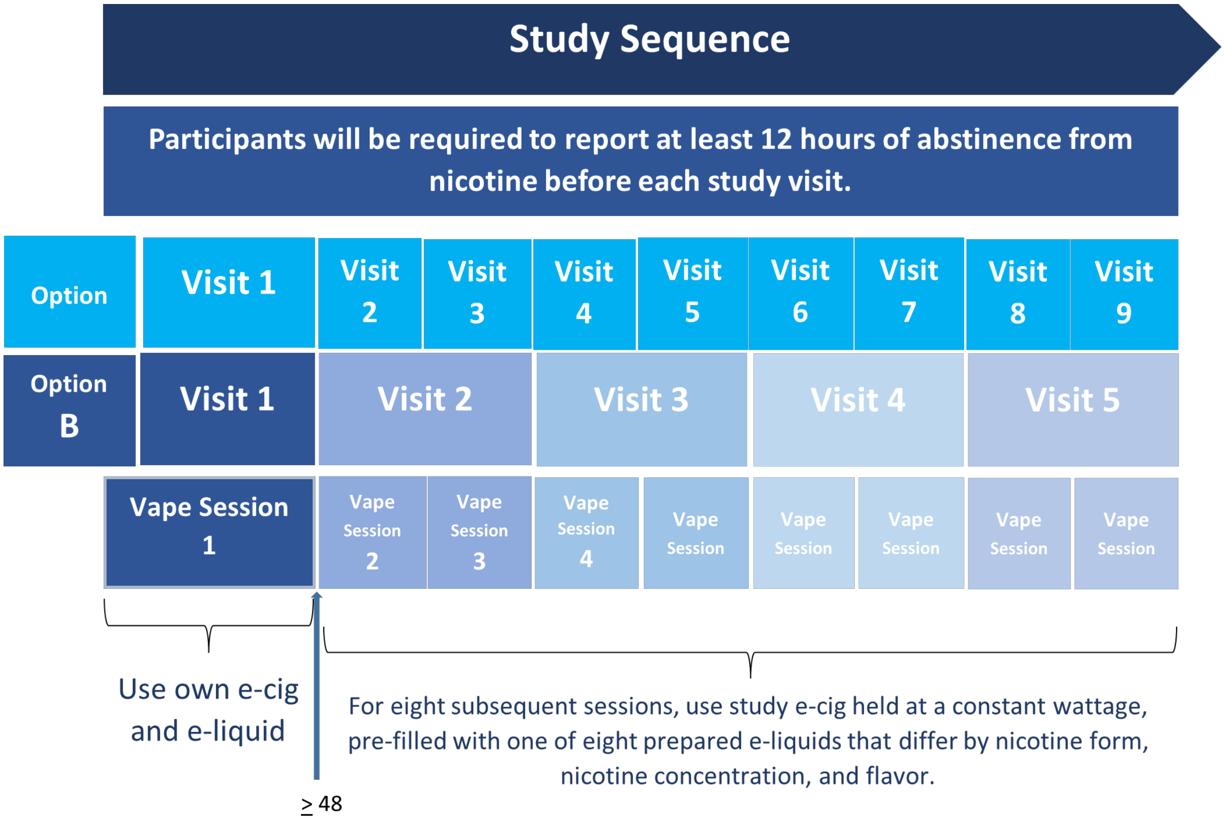

Figure 2. Study Products

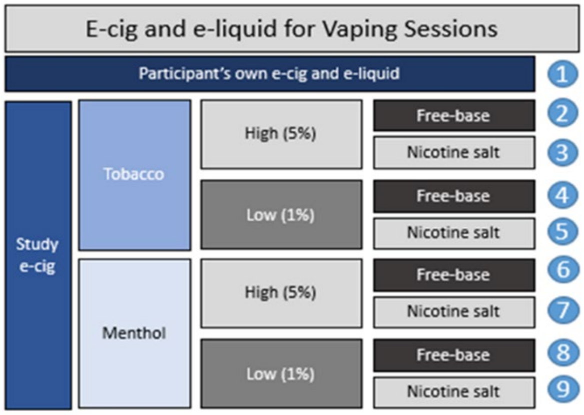

Figure 3. Vaping Session

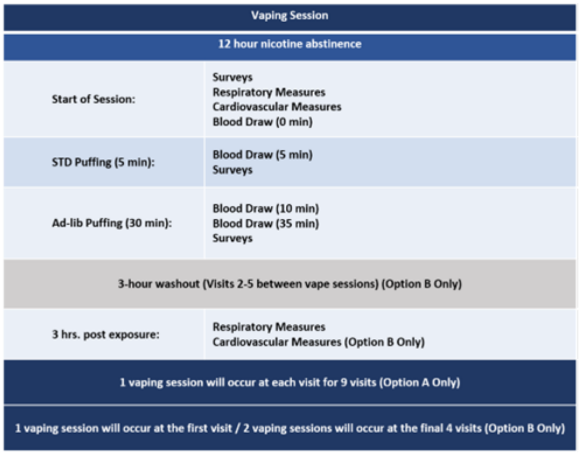

METHODS

A. Participants, interventions, outcomes

Participants. Participants who meet the following eligibility criteria will be asked to take part in the study.

Inclusion criteria: 1) a current e-cigarette user (≥1 vaping bout per day) for at least the past 3 months , 2) 21-25 years old, 3) willing to abstain from all tobacco and nicotine for at least 12 hours prior to lab sessions, 4) willing to complete nine lab visits/vaping session lasting up to 2 hours each or five lab visits lasting up to 6 hours each (except Visit 1) , 5) able to read and speak English, 6) willing to provide informed consent.

Exclusion criteria: 1) self-reported diagnosis of lung disease including asthma, cystic fibrosis, or chronic obstructive pulmonary disease, 2) history of cardiac event or distress within the past 3 months, 3) currently pregnant (determined using urine pregnancy test), planning to become pregnant, or breastfeeding, 4) use of other tobacco products >10 days in the past month, 5) current marijuana use >10 times per month, 6) currently engaging in a vaping cessation attempt.

At first contact, all participants will be screened according to the study's inclusion/exclusion criteria. Those who are eligible will be given a brief verbal overview of the study and invited to participate. Informed consent (including a description of the nature, purpose, risks, and benefits of the study) of the participant will take place through both oral and written explanation of the study. The voluntary nature of the study and the participant's right to withdraw at any time will be stressed during the consent process. A copy of the informed consent will be provided to the participant in written form at the time of consent for them to keep. Informed consent will be collected by IRB approved study personnel. Recruitment script and materials, consent forms, and all study procedures will be approved by the OSU Institutional Review Board. All participants will provide written consent before any study data is collected.

We intend to recruit 130 e-cig users from the community over an 18-month period and have them complete nine study visits (Option A) or five study visits (Option B). This study, for each of the main effects and alpha = 0.01, achieves 80% power to detect an effect size of 0.5 standard deviations of the paired differences. For interaction effects, we will estimate 80% power (effect size 0.5 SD) to distinguish between any two of the interaction groups (e.g., free base/low nic, free base/high nic), while correcting for the pairwise tests (p=0.01/6=0.0017). Carryover and period effects will also be examined.

Intervention. Participants will complete vaping sessions which will include a standardized, 5-minute, 10-puff vaping bout (30 seconds between each puff) followed by 30 minutes of ad libitum vaping. Prior to session one, participants will be asked to bring their own e-cig device and a full tank/pod to the first visit. In session one, participants will use their own e-cig and e-liquid. After visit 1, participants will take home a study e-cig device and 1 weighed pod pre-filled with study e-liquid (the participant was randomized to at the end of visit 1) to practice (20 puffs per day with the study device and until the participant feels comfortable) using the device before visit 2. Practice use with the study e-cig device and pre-filled pod will be verified based on data downloaded (via eScribe) from the device during visit 2. In the subsequent vape sessions, participants will use a study e-cig held at a constant wattage, and pre-filled with e-liquid of different nicotine form (free-base vs. nicotine salt), concentration (low-1% vs. high-5%), and flavoring (tobacco vs. menthol). All sessions will be completed over nine visits for schedule Option A and 5 visits for schedule Option B. Study Schedule A consist of 9 separate visits and 9 separate vaping sessions. Study schedule B will consist of five separate visits (each visit will

include up to 2 vaping sessions) each separated by a 3-hour washout period to allow nicotine levels to return to baseline. Each visit will be at least 48 hours apart. Participants will be required to report at least 12 hours abstinent from nicotine before each study visit. Participants will be told that their abstinence will be confirmed at the time of visit via blood plasma nicotine analysis. This is a partial bogus pipeline;[7] 3mL venous blood sample will be collected for later analysis; those with plasma nicotine levels >3ng/mL at the time of their visit will be replaced post hoc with another participant. We conservatively estimate replacing 10% of participants.

Outcomes. Measures of topography, nicotine uptake, abuse liability, subjective effects, and cardiovascular and pulmonary effects will be collected (Table 1). Exposure to select toxicants, including nicotine and menthol in total particulate matter, and gas phase carbonyls and volatile organic compounds, will also be estimated post hoc using puff playback machine smoking.

Visit	1	2-9 (Option A) or 2-5 (Option B)
Background Measures		
Sociodemographic Measures	X	
Tobacco Use History & Sensory E-Cigarette Expectancies Scale (SEES)	X	
E-Cig Dependence (modified Cigarette Dependence Scale)	X	
Timeline Followback (week, month, lifetime)	X	
E-cig Abuse Liability		
EC Puff Topography (puff count, puff duration, inter-puff interval, puff flow rate, average puff volume, total puff volume)	X	X
Subjective Effects		
Drug Effects/Liking Questionnaire	X	X
modified Cigarette Evaluation Questionnaire (mCEQ)	X	X
Sensory E-Cigarette Expectancies Scale	X	X
Behavioral Economic Demand		
E-Cigarette Purchase Task	X	X
E-cig Craving/Suppression of Craving and Withdrawal		
Tiffany-Drobes Questionnaire of Smoking Urges: Brief Form	X	X
Minnesota Nicotine Withdrawal Scale Self-Report	X	X
Nicotine Uptake		
Forearm Venous Catheter	X	X
E-liquid Consumption		
e-liquid containers for pods will be weighed	X	X
Toxicant Exposure		
Average topography “puff playback,” and e-cig aerosol produced and collected on filters	X	X
Liquid and Gas Chromatography/Mass Spectrometry	X	X
E-cig Device Characteristics		
Device voltage, coil resistance, pressure drop	X	X
E-liquid Characteristics		
pH, nicotine concentration, and PG/VG ratio	X	X
Physiological Effects (Pulmonary)		
Laboratory Spirometry (SpiroLab)	X	X
Airway Inflammation (NIOX VERO)	X	X
Airway Reactivity (TremoFlo)	X	X
Cardiovascular Measures		

Vascular Reactivity (Vicorder or similar)	X	X
Blood Pressure	X	X
Heart Rate	X	X
Markers of endothelial dependent and independent function		
B-mode ultrasound (Vicorder or similar)	X	X
Arterial stiffness		
Pulse wave velocity and analysis (Vicorder or similar)	X	X

B. Assignment of interventions

Product sampling will be randomized using a Latin Square, ensuring that each participant has an equal chance of receiving any given product. Participants will be blinded during the sampling processes.

C. Data collection, management, and analysis

Data collection and management. All research staff will have completed Human Subjects and HIPAA training. Standard operating procedures (SOP) will be developed, and all staff will be trained to ensure adherence to the SOP. As is standard practice for our team's current studies, each visit will have its own checklist of specific measures to be completed and the order in which they are to be administered. To reduce data entry errors, participants will enter data into secured computer-based questionnaires, using an electronic data capture system (REDCap). All specimens collected for biomarker analysis will be given individualized bar codes. All electronic data will be numerically coded and stored in a password protected database, on a password protected computer in a secure research space. Participant information will be accessible only to research staff, who are pledged to confidentiality and complete training in the ethical conduct of research (i.e., both HIPAA and CITI trainings). Identifying information will not be reported in any publication. All key on-site personnel will meet face-to-face weekly throughout the entire study. During these meetings, recruitment, enrollment, data collection, data monitoring results, and any concerns/issues will be discussed.

Statistical methods. Generalized linear mixed models will be employed to identify associations of nicotine form, nicotine concentration, and flavoring with various outcome measures (e.g., plasma nicotine, spirometry, flow-mediated dilation). Models will include relevant covariates (e.g., gender), a random effect for participant, main effects for nicotine form and concentration, and the interaction between nicotine form and concentration and nicotine form and flavor. Because of multiple measurements tested, we will assume a significance threshold corresponding to one expected false discovery per 100 tests (p=0.01).

Because product sampling will be randomized using a Latin Square, a fixed effect for session and a random effect for sequence will be included in all analyses.

While we will make every effort to minimize the missing data for this study, missing data can arise due to various reasons. Violation of missing complete at random (MCAR) will be checked by evaluating whether any covariates are associated with missing data. If so, these covariates will be subsequently included and controlled for in the GLMM model. Our primary analysis will use mixed models which assumes Missing At Random (MAR) to deal with the missing data problem. In addition, sensitivity analyses will also be conducted using results from multiple imputation and complete cases strategies.

D. Monitoring

Data and safety monitoring board. The data and safety monitoring plan will involve the continuous evaluation of safety, data quality and data timeliness. Investigators will conduct continuous review of data and patient safety at their regular Disease Group meetings (at least monthly). The PI of the trial will review toxicities and responses of the trial where applicable at these disease center meetings and determine if the risk/benefit ratio of the trial changes. Frequency and severity of adverse events will be reviewed by the PI and compared to what is known about the agent/device from other sources; including published literature, scientific meetings and discussions with the sponsors, to determine if the trial should be terminated before completion. Serious adverse events and responses will also be reviewed by the OSUCCC Data and Safety Monitoring Committee (DSMC). The PI will also submit a progress report (biannually for Phase II and quarterly for Phase I) that will be reviewed by the committee per the IRB of record as per the policies of the IRB. All Serious Adverse Events are to be submitted to the DSMC for their review. Submissions are made via OnCore.

Adverse Events. Adverse events will be assessed by study staff at each visit via participant self-report and managed immediately. All adverse events will be reported to the OSU IRB. We will monitor for risk of smoking/vaping by screening participants for general medical precautions (pregnancy, cardiovascular disease). Any adverse events, breaks of confidentiality, or any other data or safety issues that arise will be discussed immediately between study personnel and Dr. Wagener. Dr. Wagener will be responsible for completing an Adverse Events Form should an event occur. Dr. Wagener will report Serious Adverse Events to the OSU IRB within 24 hours of having received notice of the event. Dr. Wagener will gather any information needed to investigate the event and to determine subsequent action. Any subsequent action will be documented and reported to the OSU IRB and the Program Officer at NIH. Adverse event reports will be reviewed annually with the OSU IRB to ensure participant safety.

ETHICS

The study was approved by the Ohio State University Institutional Review Board (Study ID: 2020C0169).

REFERENCES

1. Tobacco Product Standards. 2023. https://www.fda.gov/tobacco-products/products-guidance-regulations/tobacco-product-standards
2. Leavens ELS, Stevens EM, Brett EI, Leffingwell TR, Wagener TL. JUUL in school: JUUL electronic cigarette use patterns, reasons for use, and social normative perceptions among college student ever users. *Addictive Behaviors*. 2019/12/01/ 2019;99:106047. doi:https://doi.org/10.1016/j.addbeh.2019.106047
3. Leavens ELS, Stevens EM, Brett EI, et al. JUUL electronic cigarette use patterns, other tobacco product use, and reasons for use among ever users: Results from a convenience sample. *Addictive Behaviors*. 2019/08/01/ 2019;95:178-183. doi:https://doi.org/10.1016/j.addbeh.2019.02.011
4. Brett EI, Stevens EM, Wagener TL, et al. A content analysis of JUUL discussions on social media: Using Reddit to understand patterns and perceptions of JUUL use. *Drug and Alcohol Dependence*. 2019/01/01/ 2019;194:358-362. doi:https://doi.org/10.1016/j.drugalcdep.2018.10.014
5. Brinkman M, Keller-Hamiltion B, Carney-Knisely G, et al. Effect of PH of the nicotine liquid on JUUL puuffing behavior and nicotine uptake. 2020.
6. Leavens E, Mehta T, Hale JJ, Shihadeh A, Halquist M, Wagener TL. Nicotine delivery and abuse liability of pod-mod electronic cigarettes compared to combustible cigarettes. 2020.
7. Roese NJ. Twenty years of bogus pipeline research: A critical review and meta-analysis. *Psychological bulletin*. 1993 1993;114(2):363-375. doi:10.1037/0033-2909.114.2.363
