# Supplementary material for: E-Cigarette Nicotine Delivery Among Young Adults by Nicotine Form, Concentration, and Flavor: A Crossover Randomized Clinical Trial
Source: JAMA Netw Open. 2024 Aug 9;7(8):e2426702. doi: 10.1001/jamanetworkopen.2024.26702 (PMC11316233; doi:10.1001/jamanetworkopen.2024.26702)
Supplement: Supplement 2. — eTable 1. Ingredients of the Investigational Tobacco Products eTable 2. Theoretical and Measured Ingredients in the Investigational Tobacco Products eTable 3. Participant Characteristics and Tobacco Use History by Preferred Number of Visits eTable 4. Participant Characteristics and Tobacco Use History eFigure 1. Study Device eFigure 2. Study Sequence eFigure 3. Topography During the 30-Minute Ad Libitum Vaping Session by Nicotine Concentration and Form eFigure 4. Urges and Cravings for Vaping eFigure 5. CONSORT Diagram eReference [file jamanetwopen-e2426702-s002.pdf]

Supplementary Online Content

Cho YJ, Mehta T, Hinton A, et al. e-Cigarette nicotine delivery among young adults by nicotine form, concentration, and flavor: a crossover randomized clinical trial. *JAMA Netw Open*. 2024;7(8):e2426702. doi:10.1001/jamanetworkopen.2024.26702

eTable 1. Ingredients of the Investigational Tobacco Products
eTable 2. Theoretical and Measured Ingredients in the Investigational Tobacco Products
eTable 3. Participant Characteristics and Tobacco Use History by Preferred Number of Visits
eTable 4. Participant Characteristics and Tobacco Use History
eFigure 1. Study Device
eFigure 2. Study Sequence
eFigure 3. Topography During the 30-Minute Ad Libitum Vaping Session BY Nicotine Concentration and Form
eFigure 4. Urges and Cravings for Vaping
eFigure 5. CONSORT Diagram
eReference

This supplementary material has been provided by the authors to give readers additional information about their work.

© 2024 Cho YJ et al. *JAMA Network Open*.

eTable 1. Ingredients of the Investigational Tobacco Products

ITP Name	Dominant Nicotine Form	–(-)Nicotine (mg/g)	Flavoring	Benzoic acid (mg/g)	Glycerol (mg/g)	Propylene Glycol (mg/g)
T5FB	Freebase	50	Tobacco (Ghost Town[b])	None	700	300
T5NS	Salt-based[a]			37.65		
T1FB	Freebase	10		None		
T1NS	Salt-based			7.53		
M5FB	Freebase	50	Menthol (Yeti[a])	None	700	300
M5NS	Salt-based			37.65		
M1FB	Freebase	10		None		
M1NS	Salt-based			7.53		

[a] Nicotine was protonated with a 2% molar excess of benzoic acid

[b] Ghost Town and Yeti are brand names for the ITPs provided to us by Flagship Vapor.

© 2024 Cho YJ et al. *JAMA Network Open*.

eTable 2. Theoretical and measured ingredients in the investigational tobacco products; mean (standard deviation) for replicates (n=3) except for freebase fraction (n=1 pH determination)

Sample ID	Nicotine (% w/w)[a,b]		Freebase Nicotine (% of Total Nicotine)[c]		Propylene Glycol (% w/w)[b]		Glycerol (% w/w)[b]		Menthol (% w/w)[b]	
	Theoretical	Measured	Theoretical	Measured	Theoretical	Measured	Theoretical	Measured	Theoretical	Measured
T5FB	5.00	5.05 (0.02)	100	98.1	30.00	31.35 (0.16)	60.00	59.53 (0.14)	0	ND
T5NS	5.00	4.99 (0.05)	0	0.3	30.00	29.87 (0.20)	60.00	56.19 (0.37)	0	ND
T1FB	1.00	0.97 (0.00)	100	94.3	30.00	32.52 (0.12)	60.00	61.71 (0.48)	0	ND
T1NS	1.00	0.97 (0.00)	0	0.2	30.00	32.16 (0.13)	60.00	60.44 (0.22)	0	ND
M5FB	5.00	5.09 (0.02)	100	98.6	30.00	28.63 (0.10)	60.00	59.96 (0.18)	1.00	0.93 (0.00)
M5NS	5.00	5.05 (0.02)	0	0.2	30.00	27.17 (0.14)	60.00	56.74 (0.22)	1.00	0.83 (0.00)
M1FB	1.00	1.01 (0.00)	100	95.7	30.00	29.88 (0.22)	60.00	62.45 (0.20)	1.00	0.97 (0.00)
M1NS	1.00	0.98 (0.00)	0	0.3	30.00	29.47 (0.08)	60.00	61.55 (0.26)	1.00	0.70 (0.03)

[a] % (w/w) = weight of chemical / weight of e-liquid.

[b] Analyzed using quantitative nuclear magnetic resonance as described in Crenshaw et al., 2016.[1]

[c] 10g of HPLC grade deionized water was added to 1g of e-liquid solution. The mixture was stirred for 15 minutes and the pH was measured. The freebase fraction was calculated using the Henderson-Hasselbalch equation.

ND = not detected

© 2024 Cho YJ et al. *JAMA Network Open*.

eTable 3. Participant Characteristics and Tobacco Use History by Number of Visit Preference

	**5 Visits** (n=21) Mean (SD) or No. (%)	**9 Visits** (n=66) Mean (SD) or No. (%)	p-value
Age	21.9 (1.0)	22.5 (1.4)	0.05
Female sex assigned at birth	12 (57.1%)	39 (59.1%)	0.87
Race/ethnicity[a]			0.91
Hispanic	1 (4.8%)	5 (7.6%)	
Non-Hispanic Black	1 (4.8%)	3 (4.6%)	
Non-Hispanic White	16 (76.2%)	52 (78.8%)	
Other (e.g., multiple races)	3 (14.3%)	6 (9.1%)	
E-cigarette use characteristics			
Days of use during the past 30 days	28.6 (3.2)	27.0 (6.8)	0.71
Years of regular e-cigarette use[b]	3.4 (2.2)	3.3 (2.1)	0.74
Current regular e-cigarette use[b]	21 (100.0%)	63 (96.9%)	1.00
Average Puffs per Day	68.8 (42.3)	82.8 (104.6)	0.74
Flavor[c]			0.38
Tobacco	0 (0.0%)	0 (0.0%)	
Menthol or Mint	10 (47.6%)	24 (36.9%)	
Other flavor	11 (52.4%)	41 (63.1%)	
Nicotine Form[c]			0.59
Nicotine Salts	15 (71.4%)	51 (77.3%)	
Unknown	6 (28.6%)	15 (22.7%)	
Nicotine Concentration, %[c]			
Other tobacco use characteristics			
Ever smoking of a cigarette	20 (95.2%)	55 (83.3%)	0.28
Ever regular cigarette smoking[a]	4 (19.1%)	16 (24.6%)	0.77
Ever use of a cigar	14 (66.7%)	31 (47.7%)	0.13
Ever use of a pipe	5 (23.8%)	11 (16.9%)	0.52
Ever use of cigarillos	5 (23.8%)	30 (46.2%)	0.07
Ever use of little cigars	2 (9.5%)	7 (10.8%)	1.00
Ever use of smokeless tobacco	5 (23.8%)	17 (26.2%)	0.83
Ever use of snus	0 (0.0%)	6 (9.2%)	0.33
Ever use of hookah	11 (52.4%)	36 (55.4%)	0.81

[a]Ethnicity was self-reported as 'Hispanic or Latino' or 'Not Hispanic or Latino'. Race was self-reported as 'American Indian/Alaskan Native,' 'Asian,' 'Black or African American,' 'Native Hawaiian or Other Pacific Islander,' 'White or Caucasian,' and 'Other,' with multiple choices allowed. The 'Other' category includes non-Hispanic American Indian, Asian, and Pacific Islander. [b]The regular use of a product was defined as using it for at least once a week, for at least a month. [c]Characteristics of e-liquids brought to visit 1. The research staff determined the nicotine form by examining pictures of the participants' usual e-cigarette brands. Brands using cartridges or pods filled with nicotine salts, such as JUUL, Hyde, Elf bar and Breeze Pro, were categorized as nicotine salts, whereas devices designed for user-supplied e-liquid, such as Vaporesso and Caliburn, were categorized as unknown.

© 2024 Cho YJ et al. *JAMA Network Open.*

eTable 4. Participant Characteristics and Tobacco Use History

	All Enrolled (n=87) Mean (SD) or No. (%)	Used study e-liquids[a] (n=72) Mean (SD) or No. (%)	Did not use study e-liquids[a] (n=15) Mean (SD) or No. (%)	p-value
Age	22.4 (1.4)	22.4 (1.4)	22.3 (1.1)	0.78
Female sex assigned at birth	51 (58.6%)	42 (58.3%)	9 (60.0%)	0.91
Race/ethnicity[b]				0.77
Hispanic	6 (6.9%)	6 (8.3%)	0 (0.0%)	
Non-Hispanic Black	4 (4.6%)	3 (4.2%)	1 (6.7%)	
Non-Hispanic White	68 (78.2%)	55 (76.4%)	13 (86.7%)	
Other (e.g., multiple races)	9 (10.3%)	8 (11.1%)	1 (6.7%)	
E-cigarette use characteristics				
Days of use during the past 30 days	27.4 (6.2)	27.4 (6.2)	27.4 (6.1)	0.88
Years of regular e-cigarette use[c]	3.3 (2.1)	3.3 (2.2)	3.2 (1.6)	0.88
Current regular e-cigarette use[c]	84 (97.7%)	71 (98.6%)	13 (92.9%)	0.30
Average Puffs per Day	79.4 (93.4)	85.8 (101.0)	48.9 (26.4)	0.09
Flavor[d]				0.13
Tobacco	0 (0.0%)	0 (0.0%)	0 (0.0%)	
Menthol or Mint	34 (39.5%)	31 (43.1%)	3 (21.4%)	
Other flavor	52 (60.5%)	41 (56.9%)	11 (78.6%)	
Nicotine Form[d]				1.00
Nicotine Salts	66 (75.9%)	54 (75.0%)	12 (80.0%)	
Unknown	21 (24.1%)	18 (25.0%	3 (20.0%)	
Nicotine Concentration, %[c]	4.4 (1.3)	4.5 (1.2)	4.2 (1.5)	0.53
Other tobacco use characteristics				
Ever smoking of a cigarette	75 (86.2%)	64 (88.9%)	11 (73.3%)	0.21
Ever regular cigarette smoking[c]	20 (23.3%)	17 (23.6%)	3 (21.4%)	1.00
Ever use of a cigar	45 (52.3%)	39 (54.2%)	6 (42.9%)	0.56
Ever use of a pipe	16 (18.6%)	15 (20.8%)	1 (7.1%)	0.45
Ever use of cigarillos	35 (40.7%)	30 (41.7%)	5 (35.7%)	0.77
Ever use of little cigars	9 (10.5%)	9 (12.5%)	0 (0.0%)	0.34
Ever use of smokeless tobacco	22 (25.6%)	19 (26.4%)	3 (21.4%)	1.00
Ever use of snus	6 (7.0%)	6 (8.3%)	0 (0%)	0.58
Ever use of hookah	47 (54.7%)	39 (54.2%)	8 (57.1%)	1.00
Number of Visits				0.51
5 Visits	21 (24.1%)	19 (26.4%)	2 (13.3%)	
9 Visits	66 (75.9%)	53 (73.6%)	13 (86.7%)	

[a]72 participants completed the second visit and used at least one study e-liquid, comprising the analytic sample. 15 participants completed only a baseline visit and did not try any study e-liquids. [b]Ethnicity was self-reported as 'Hispanic or Latino' or 'Not Hispanic or Latino'. Race was self-reported as 'American Indian/Alaskan Native,' 'Asian,' 'Black or African American,' 'Native Hawaiian or Other Pacific Islander,' 'White or Caucasian,' and 'Other,' with multiple choices allowed. The 'Other' category includes non-Hispanic American Indian, Asian, and Pacific Islander. [c]The regular use of a product was defined as using it for at least once a week, for at least a month. [d]Characteristics of e-liquids brought to visit 1. The research staff determined the nicotine form by examining pictures of the participants' usual e-cigarette brands. Brands using cartridges or pods filled with nicotine salts, such as JUUL, Hyde, Elf bar and Breeze Pro, were categorized as nicotine salts, whereas devices designed for user-supplied e-liquid, such as Vaporesso and Caliburn, were categorized as unknown.

© 2024 Cho YJ et al. *JAMA Network Open*.

eFigure 1. Study device (Evolv Reflex)

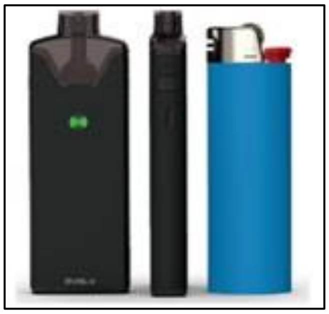

The Evolv Reflex was selected for its ability to vape lab-prepared e-liquids and its design similarity to currently available cartridge/pod type e-cigarette devices. The device has been fully characterized and includes puff actuation, 750 mAH battery, 3-25 watt range, 0.5 ohm coil resistance, and 2 mL pod capacity. Emissions testing demonstrated that its nicotine yield under normal use conditions was comparable to typical cigarette yields.

© 2024 Cho YJ et al. *JAMA Network Open*.

eFigure 2. Study sequence

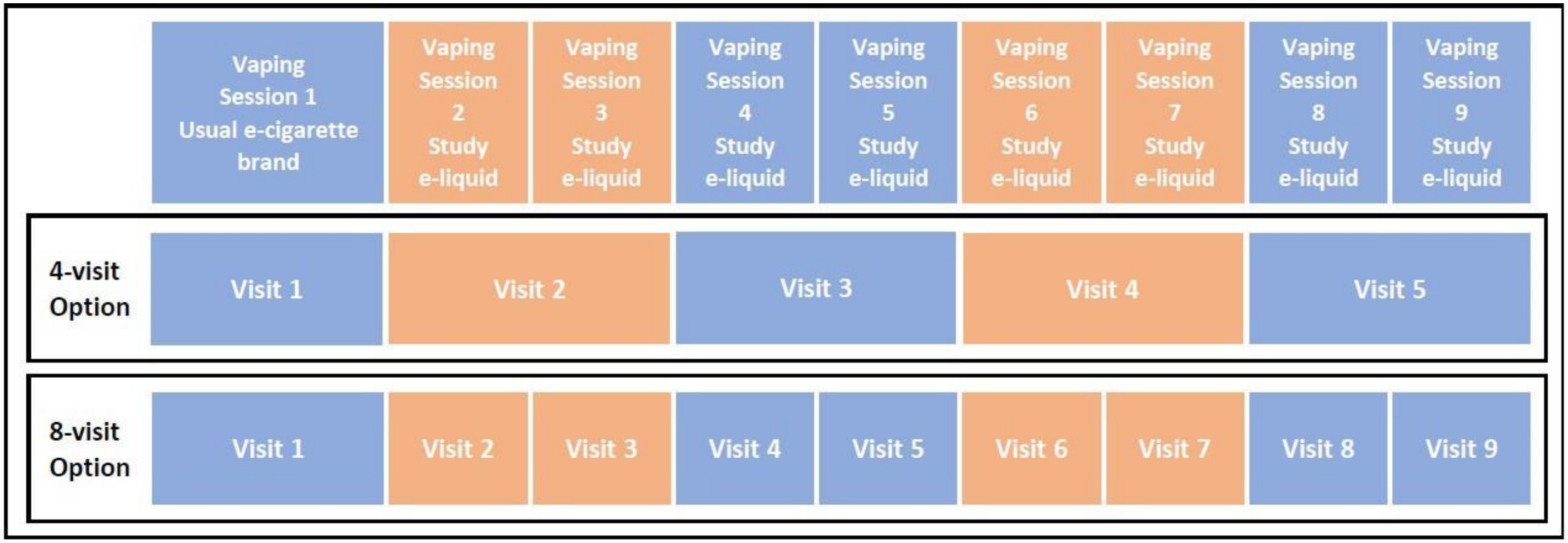

Note: Participants were required to abstain from nicotine for at least 12 hours before each study visit, with visits separated by a minimum of 48 hours. During the first visit, participants used their usual brand of e-cigarettes. Participants were then given two options for completing the subsequent eight vaping sessions: either in four or eight lab visits based on their preference. For these sessions, the Evolv Reflex e-cigarette device was used, which was set to a constant constant wattage of 8W, and prefilled with one of the eight study e-liquids. The e-liquids varied in nicotine form (freebase vs. salt-based nicotine), nicotine concentration (1% vs. 5% w/w), and flavor (menthol vs. tobacco) and were administered in a randomly assigned order.

© 2024 Cho YJ et al. *JAMA Network Open*.

eFigure 3. Topography during the 30-minute ad libitum vaping session by nicotine concentration and form.

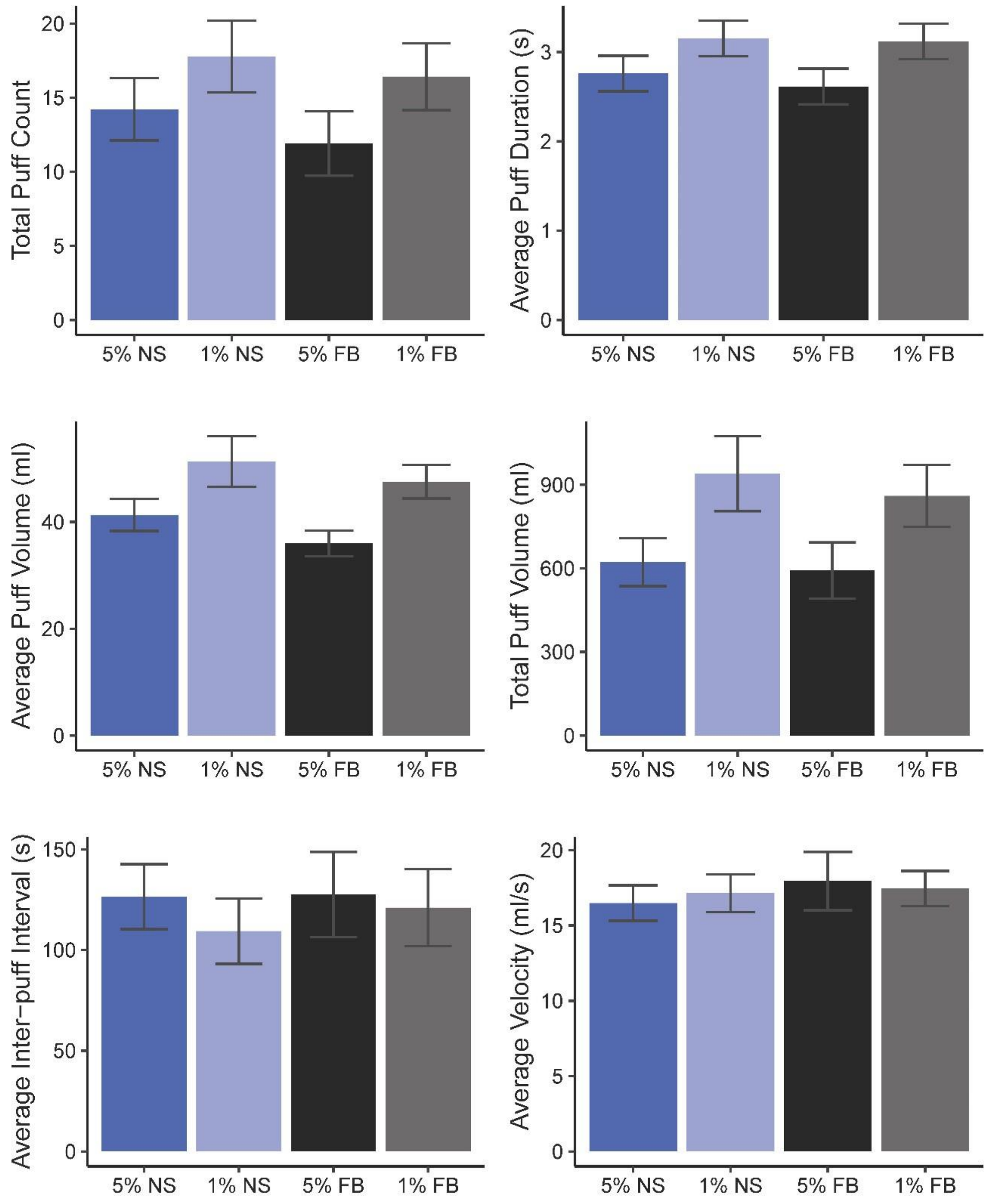

VAS: visual analog scale; NS: nicotine salts; FB: freebase nicotine. The boxes represent the means, and the lines represent the 95% confidence intervals. The unit measures were n for total puff count, seconds for average puff duration, and milliliters (ml) for the average puff volume and total puff volume.

© 2024 Cho YJ et al. *JAMA Network Open*.

eFigure 4. Urges and cravings for vaping

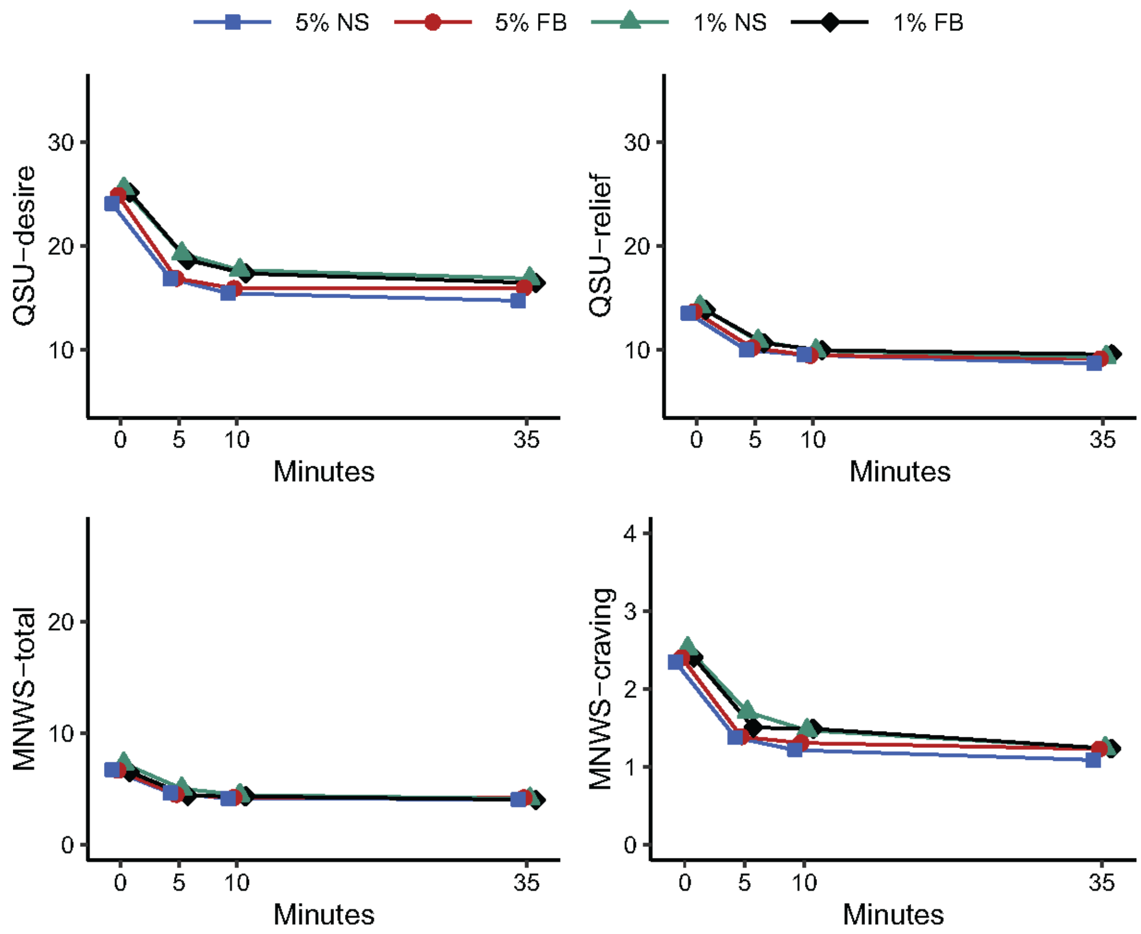

QSU: Questionnaire of Smoking Urges; MNWS: Minnesota Nicotine Withdrawal Scale. QSU-desire and QSU-relief ranged from 1 to 35. MNWS-total ranged from 0 to 32. MNWS-craving ranged from 0 to 4.

© 2024 Cho YJ et al. *JAMA Network Open*.

eFigure 5. CONSORT diagram

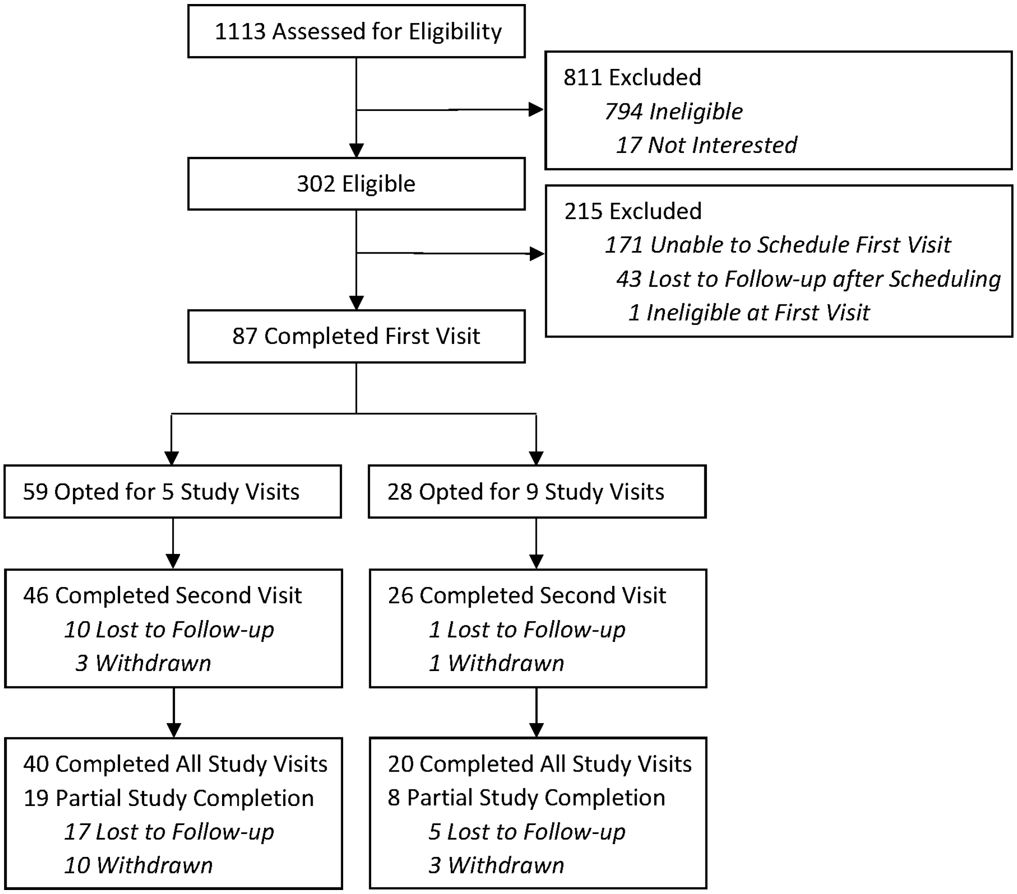

© 2024 Cho YJ et al. *JAMA Network Open*.

eReference

1. Crenshaw MD, Tefft ME, Buehler SS, Brinkman MC, Clark PI, Gordon SM. Determination of nicotine, glycerol, propylene glycol and water in electronic cigarette fluids using quantitative (1) H NMR. *Magn Reson Chem*. Nov 2016;54(11):901-904. doi:10.1002/mrc.4498

© 2024 Cho YJ et al. *JAMA Network Open*.
