# Supplementary material for: E-Cigarette Nicotine Delivery Among Young Adults by Nicotine Form, Concentration, and Flavor: A Crossover Randomized Clinical Trial
Source: JAMA Netw Open. 2024 Aug 9;7(8):e2426702. doi: 10.1001/jamanetworkopen.2024.26702 (PMC11316233; doi:10.1001/jamanetworkopen.2024.26702)
Supplement: Supplement 3. — Data Sharing Statement [file jamanetwopen-e2426702-s003.pdf]

Data Sharing Statement

Cho. e-Cigarette Nicotine Delivery Among Young Adults by Nicotine Form, Concentration, and Flavor. *JAMA Netw Open*. Published August 09, 2024.
doi:10.1001/jamanetworkopen.2024.26702

Data
Data available: No
